# Supplementary figures and images for: Reversing a reported case of transoceanic dispersal: Nudibranch identifications among tsunami debris
Source: PLoS One. 2024 Dec 12;19(12):e0306586. doi: 10.1371/journal.pone.0306586 (PMC11637273; doi:10.1371/journal.pone.0306586)

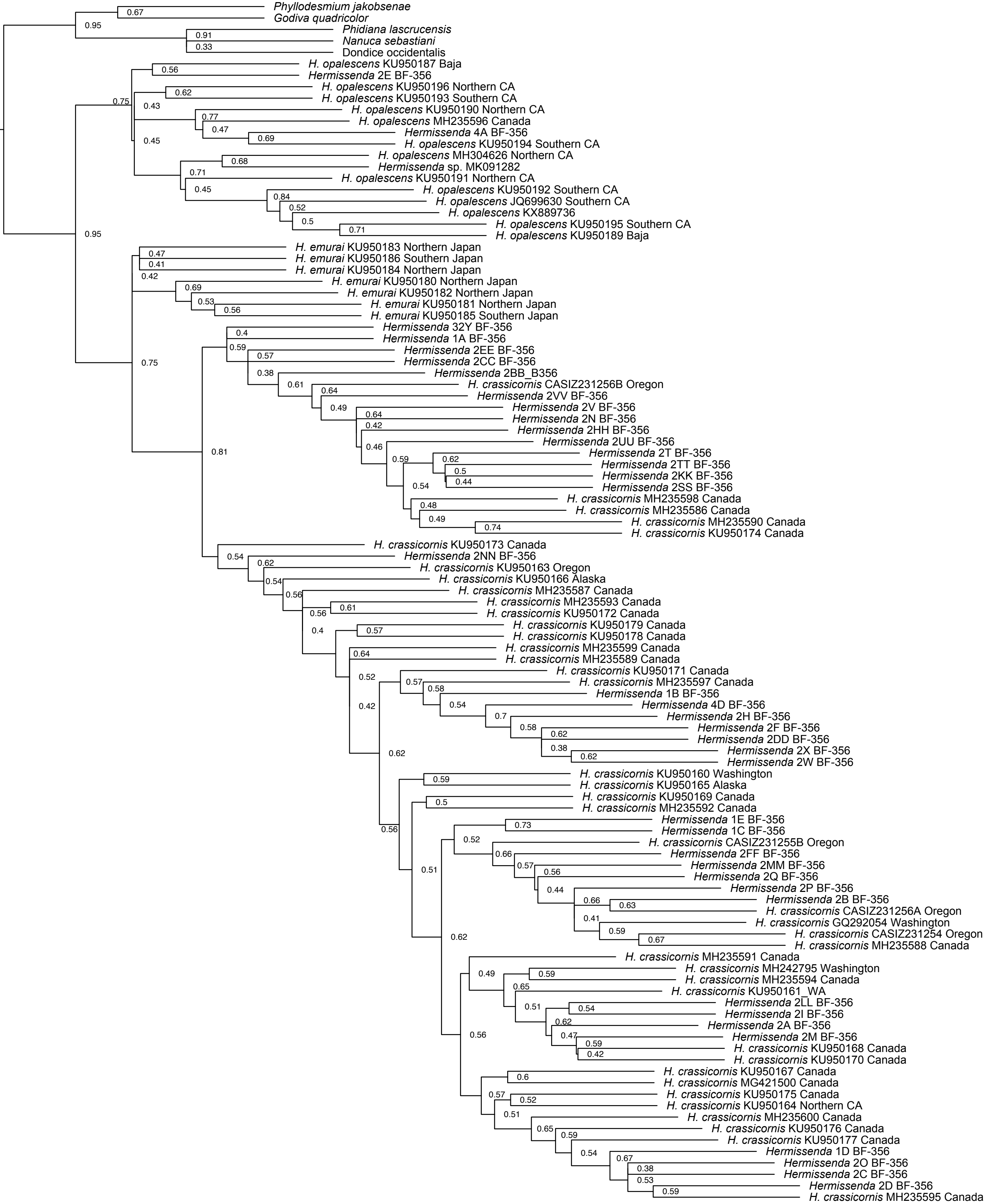

0.7

Supplement: S1 Fig — (PDF) [file pone.0306586.s001.pdf]
