# Supplementary material for: Reversing a reported case of transoceanic dispersal: Nudibranch identifications among tsunami debris
Source: PLoS One. 2024 Dec 12;19(12):e0306586. doi: 10.1371/journal.pone.0306586 (PMC11637273; doi:10.1371/journal.pone.0306586)
Supplement: S3 Table — (DOCX) [file pone.0306586.s004.docx]

|  | *H. opalescens* | *H. crassicornis* | *H. emurai* |
| --- | --- | --- | --- |
| *H. opalescens* | 0–1.36% |  |  |
| *H. crassicornis* | 3.44%–5.17% | 0–0.93% |  |
| *H. emurai* | 3.42%–5.21% | 2.00%–3.40% | 0.46–1.67% |
