# Supplementary material for: Reversing a reported case of transoceanic dispersal: Nudibranch identifications among tsunami debris
Source: PLoS One. 2024 Dec 12;19(12):e0306586. doi: 10.1371/journal.pone.0306586 (PMC11637273; doi:10.1371/journal.pone.0306586)
Supplement: Attachment — Submitted filename: Review of Montana el al for PLoS One.pdf [file pone.0306586.s005.pdf]

TITLE: Why didn't the nudibranch cross the ocean? Understanding biogeographic and evolutionary replationships of *Hermissenda* (Nudibranchia: Myrrhinidae) Bergh, 1878

AUTHORS: Montana, Gosliner, Crews, Bonomo, Carlton and Johnson

GENERAL REMARKS:

This manuscript is addressed contribute for a better understanding of the effects of the tsunami happened in 2011 in Japan concerning the transportation/invasion of organisms from western to eastern Pacifc coasts. Thus, the study is focussed on the species of the aeolid nudibranch genus *Hermissenda* (*H. crassicornis* and *H. opalescenes*) found on a vessel from Iwate Prefecture (later designated as JTMD-BF-356) discoverd at the coast of Oregon in april 2015. A good number of non native invertebrate species were found and reported previoussly on the surface of this vessel. Thus, among the nudibranch individuals found, stand out two especies: *Dendronotus venustus* and *Eubranchus rustyus*, both only known fro western Pacific. However, the individuals of the genus Hermissenda belongs to H. crassicornis and H. opalescens, both known to date to eastern Pacific only. No individuals of *H. emurai* (from western Pacific) were found among the vessel *Hermissenda* individuals. Then, two hypothesis arise to try to explain the presence of the above species: or they were settled after the arrival of the vessel or both species are present in Japanese coasts, but this presence were unnoticed to date.

Taxonomical identification of *Hermissenda* species is confirmed by molecular phylogenetic and haplotype studies, complemented by a delimitation species análisis.

In general terms, I agree with the conclusions of the manuscript. Nevertheless, I have several concerns that should be corrected/clarified/discussed before the manuscript could be published (see below).

PARTICULAR REMARKS:

Title: place "Bergh, 1879" after *Hermissenda*. Moreover, the date of the genus is 1879, instead 1878 (see https://www.marinespecies.org/aphia.php?p=taxdetails&id=367881)

Abstract:

Lines 42-44: "Our phylogenetic tree...*H. opalescens* and *H. emurai*". I don't understad the meaning of this paragraph. Please, check and re-write it.

Lines 41 and 44-46: I think that the reference to iNaturalist should be linked with the final paragraph (44-46). Please, re-write again.

Introduction:

Line 57: modify as "...Megina & Cervera, 2003; Nakano & Hirose, 2011) or even eggs of other nudibranchs or fishes (Todd, 1981; Calado & Urgorri, 2002)."

Line 61: modify as "(Hadfield, 1963; Todd, 1981)".

Line 82: change "family *Hermissenda*" by "genus *Hermissenda*".

Methods:

a) <u>Vessel JTMD-BF-356</u>

Line 136: change "(Carlton et al. 2017; Carlton et al. 2018…" by "Carlton et al., 2017, 2018,…"

b) Molecular analyses of nudibranchs

Why the authors have used the COI sequences in their analyses only? Lindsay and Valdés (2016) used two mitochondrial (COI, 16S) and two nuclear markers (18S, H3) and their resulting phylogenetic has its nodes well supported. Their phylogenetic hypothesis is the same of that presented by Montana et al (this manuscript), but in this last case only the monophyly of the genus Hermissenda is well supported, although the suggested/pointed relationships amomg species are similar to that of Lindsay and Valdés (2016). I suggest to inclued more molecular markers to get well supported nodes in the phylogenetic tree.

On the other hand, values to consider nodes well supported for each analysis are not indicated. Nor in the text nor the figure captions of figs 3 and 4. Please, do it.

Results:

a) JTMD-BF-356 biota

Line 196: change "fish" by "fishes".

Line 196: change "Carlton et al. 2017; Carlton et al. 2018" by "Carlton et al., 2017, 2018".

b) Molecular analyses and phylogenetic replationships of *Hermissenda*

Please, see what I have remarked in the Methods section concerning to the molecular analyses of nudibranch that refers to the support of the clades.

Lines 235-240: the authors refers to the number of denticles of the radular central cusp of the diferent species of *Hermissenda*, and also refers to the infomation supplied by Lindsay and Valdés (2016). However, I think the authors of the manuscript have been confused. Lindsay and Valdés (2016) didn't find significant radular differences among the rádula of the three species of *Hermissenda*. However, they found slightly morphological differences among the denticulation of the masticatory border of the jaws. Then, part of this paragraph needs to be rethought and rewritten again.

The authors supply radular information, but not that concerning to jaws that could be relevant (to differentiete species in this genus) according to Lindsay and Valdés (2016) stated.

Nevertheless, in my opinion, the number of examined specimens both by Linsay and Valdés (2016) and in the present manuscript are are not enough to have a clear idea about the relevance of the differences of the radulae and jaws among *H. crassicornis*, *H. opalecens* and *H. emurai*. I would check this much better...and the authors have enough material to carry on this.

There are no differenecs among the reproductive systems of the three species? Lindsay and Valdés (2016) didn't refer to this.

Line 240: "Lindsay and Valdés, 2016". Here the authors use "and" instead "&". Please, check all the text to homogenize this in all references.

Discussion:

Lines 296-306: please, see what I have remarked in the above section (Results) concerning to the study of radula and jaws.

Lines 323-339: this paragraph is very interesting because concerns to the feeding and larval biology. Thus, the authors refers to Kincaid and de Rivera (2021) report that *H. crassicornis* feeds preferently on the indigenous ascidian *Distapla occidentalis* than to the non-indigenous *Botrylloides violaceus* or *B. schlosseri*. Surprisingly, Kincais and de Rivera overlooked the previous paper of Megina et al (2007) describing the trophic strategies of *Hermissenda crassicornis*. The individuals attributed to *H. crassicornis* were collected south to San Francisco Bay and and Monterey. So, probably were specimens of *H. opalescens*, according to the range proposed in Lindsay and Valdés (2016). Megina et al (2007) observed that the main prey of *H. "crassicornis"* was *Aplidium "solidum"*. However, the nomenclature of this species was revised Brunetti (2007) and the Californian called "*solidum*" is *A. kottae* Ritter and Forsyth, 1917.

It would be very interesting/useful to know if *Aplidium kottae* was settled on the JTMD-BF-356 vessel. However, no detailed information on Tunicates is included in Carlton et al 2017 and 2018. Maybe Prof. James T. Carlton could have this information.

In any case, both *Hermissenda crassicornis* and *H. opalescens* seems to prey on indigenous tunicates preferently (*Distapla occidentalis* and *Aplidium kottae*, respectively), but not exclusively. Moreover, at least *H. opalescens* can feed on hydrozoans or antozoans probably as a source of nematocist (Megina et al, 2007). So, I think that it is much probable that *Hermissenda* populations of the JTMD-BF-356 vessel settled afeter its arrival to the Oregon coast. But, I agree that further investigations have to be carried to exclude the alternative hypothesis (presence of *H. crassicornis* and *H. opalescens* in Japanese coasts).

Acknowledgements:

Line 386: change "Hermissenda" by "*Hermissenda*" (cursive)

References cited in this review and not included in the Bibliography:

- Brunetti, R. 2007. Nomenclatural Acts: homonymy in the Ascidiacea (Tunicata) and proposed nomina nova. *Zootaxa*, 1613: 67-68.

- Calado, G. and Urgorri, V. 2002. A new species of *Calma* Alder & Hancock, 1855 (Gastropoda: Nudibranchia) with a review of the genus. *Journal of Molluscan Studies*, 68: 311-317.

- Megina, C., Gosliner, T.M. and Cervera, J.L. 2007. The use of trophic resources by a generalist eolid nudibranch: *Hermissenda crassicornis* (Mollusca: Gastropoda). *Cahiers de Biologie Marine*, 48: 1-7

- Todd, C.D. 1981. The ecology of nudibranchs molluscs. *Oceanography and Marine Biology: An Annual Review*, 19: 131-234.
