# Supplementary material for: Reversing a reported case of transoceanic dispersal: Nudibranch identifications among tsunami debris
Source: PLoS One. 2024 Dec 12;19(12):e0306586. doi: 10.1371/journal.pone.0306586 (PMC11637273; doi:10.1371/journal.pone.0306586)
Supplement: Attachment — Submitted filename: Hermissenda_response_to_reviewers.docx [file pone.0306586.s006.docx]

Dear Editorial Board,

Below you can find our responses to each revision suggestion.

Thank you very much for considering our revised manuscript for publication in *PLOS ONE*.

Kind regards,

Katherine Montana

Journal Requirements:

When submitting your revision, we need you to address these additional requirements.

1. Please ensure that your manuscript meets PLOS ONE's style requirements, including those for file naming. The PLOS ONE style templates can be found at
<https://url.avanan.click/v2/___https://journals.plos.org/plosone/s/file?id=wjVg/PLOSOne_formatting_sample_main_body.pdf___.YXAzOmNhbGFjYWRlbXk6YTpnOjA1NmVlZTA1ZDJmMzJiMTZkOGU1NDQ1YWYzMTc0OTZiOjY6MWNjYjpjNzc2OTYyOTE0N2VmMjZiZjkxOWVlYjkyOWI2YjM4NmVlMzYxNTJkZDJkY2ZkZDc4YTFkZTUxYjRlZjhjYzM4OnQ6VDpO> and
<https://url.avanan.click/v2/___https://journals.plos.org/plosone/s/file?id=ba62/PLOSOne_formatting_sample_title_authors_affiliations.pdf___.YXAzOmNhbGFjYWRlbXk6YTpnOjA1NmVlZTA1ZDJmMzJiMTZkOGU1NDQ1YWYzMTc0OTZiOjY6NmUyOTo0ZTVkNjcwZWFhOWM2NGQ4NTQ5MGVhZDAxYTJjZDJjZTdmZWEyYjU5NTRlZjg3NDEzMjY4NmY3NzRhZDc5NGMzOnQ6VDpO>

We have reviewed the style templates at the links above and ensured the manuscript meets their requirements.

2. Please note that funding information should not appear in any section or other areas of your manuscript. We will only publish funding information present in the Funding Statement section of the online submission form. Please remove any funding-related text from the manuscript.

We have removed funding information from the acknowledgements section.

3. When completing the data availability statement of the submission form, you indicated that you will make your data available on acceptance. We strongly recommend all authors decide on a data sharing plan before acceptance, as the process can be lengthy and hold up publication timelines. Please note that, though access restrictions are acceptable now, your entire data will need to be made freely accessible if your manuscript is accepted for publication. This policy applies to all data except where public deposition would breach compliance with the protocol approved by your research ethics board. If you are unable to adhere to our open data policy, please kindly revise your statement to explain your reasoning and we will seek the editor's input on an exemption. Please be assured that, once you have provided your new statement, the assessment of your exemption will not hold up the peer review process.

Our data are genetic sequences distributed via GenBank. They are already uploaded, just awaiting to be shared publicly upon publication of this paper.

4. Please include your full ethics statement in the ‘Methods’ section of your manuscript file. In your statement, please include the full name of the IRB or ethics committee who approved or waived your study, as well as whether or not you obtained informed written or verbal consent. If consent was waived for your study, please include this information in your statement as well.

We have included an ethics statement (lines 141–145); though, no IRB or ethics committee was required. We currently do not have the particular scientific collecting permit number for the more recently collected *Hermissenda* nudibranchs. The California Academy of Sciences has a policy that no specimens may enter the collection without proper documentation, so we are confident these specimens are in compliance. It is a matter of getting in contact with the person who has the permit information, and we will provide it to *PLOS ONE* as soon as we have it.

5. We note that Figure 2 in your submission contain copyrighted images. All PLOS content is published under the Creative Commons Attribution License (CC BY 4.0), which means that the manuscript, images, and Supporting Information files will be freely available online, and any third party is permitted to access, download, copy, distribute, and use these materials in any way, even commercially, with proper attribution. For more information, see our copyright guidelines: <https://url.avanan.click/v2/___http://journals.plos.org/plosone/s/licenses-and-copyright___.YXAzOmNhbGFjYWRlbXk6YTpnOjA1NmVlZTA1ZDJmMzJiMTZkOGU1NDQ1YWYzMTc0OTZiOjY6MzgyNDo2OTEzOTc5MTBiN2JmYjljNTQ1NmMzNTMzMDRjYjNhYzg1NjJlYjM3YTAyODVlMzczYjEzYTg4MWZiYjE5NDA1OnQ6VDpO>.
 We require you to either (a) present written permission from the copyright holder to publish these figures specifically under the CC BY 4.0 license, or (b) remove the figures from your submission:
 a. You may seek permission from the original copyright holder of Figure 2 to publish the content specifically under the CC BY 4.0 license.
 We recommend that you contact the original copyright holder with the Content Permission Form (<https://url.avanan.click/v2/___http://journals.plos.org/plosone/s/file?id=7c09/content-permission-form.pdf___.YXAzOmNhbGFjYWRlbXk6YTpnOjA1NmVlZTA1ZDJmMzJiMTZkOGU1NDQ1YWYzMTc0OTZiOjY6Y2M3MzoxZWI2NjNhNDJlY2Q3OGU0NTY0OGY2MDMzODEwNzdmNzljMWY2YjZmMmQ1NTllM2JmZmQ1MzY1MzMwZWNmYTcwOnQ6VDpO>) and the following text:
“I request permission for the open-access journal PLOS ONE to publish XXX under the Creative Commons Attribution License (CCAL) CC BY 4.0 (<https://url.avanan.click/v2/___http://creativecommons.org/licenses/by/4.0/___.YXAzOmNhbGFjYWRlbXk6YTpnOjA1NmVlZTA1ZDJmMzJiMTZkOGU1NDQ1YWYzMTc0OTZiOjY6OWEyMTpkMTM2MzEwN2EyMTVhZjI5M2JiZjc4NWJkNDIwYWM3N2E4MzhlOTdiNmUzZjBiNDZhMzFhNDNhZWUxODk4OGFmOnQ6VDpO>). Please be aware that this license allows unrestricted use and distribution, even commercially , by third parties. Please reply and provide explicit written permission to publish XXX under a CC BY license and complete the attached form.”

Please upload the completed Content Permission Form or other proof of granted permissions as an "Other" file with your submission.
 In the figure caption of the copyrighted figure, please include the following text: “Reprinted from [ref] under a CC BY license, with permission from [name of publisher], original copyright [original copyright year].”
 b. If you are unable to obtain permission from the original copyright holder to publish these figures under the CC BY 4.0 license or if the copyright holder’s requirements are incompatible with the CC BY 4.0 license, please either i) remove the figure or ii) supply a replacement figure that complies with the CC BY 4.0 license. Please check copyright information on all replacement figures and update the figure caption with source information. If applicable, please specify in the figure caption text when a figure is similar but not identical to the original image and is therefore for illustrative purposes only.

We have written permission from the photographer John W. Chapman, via the form you provided, that is attached to this resubmission.

6. We note that Figure 7 in your submission contain map/satellite images which may be copyrighted. All PLOS content is published under the Creative Commons Attribution License (CC BY 4.0), which means that the manuscript, images, and Supporting Information files will be freely available online, and any third party is permitted to access, download, copy, distribute, and use these materials in any way, even commercially, with proper attribution. For these reasons, we cannot publish previously copyrighted maps or satellite images created using proprietary data, such as Google software (Google Maps, Street View, and Earth). For more information, see our copyright guidelines: <https://url.avanan.click/v2/___http://journals.plos.org/plosone/s/licenses-and-copyright___.YXAzOmNhbGFjYWRlbXk6YTpnOjA1NmVlZTA1ZDJmMzJiMTZkOGU1NDQ1YWYzMTc0OTZiOjY6NWEyZjpkOGZiNGM3M2FkYzY3NzBhYTA4YTc3ZmMxNTkxM2JlZmU5MjVmNGZkMDJhYzE3NWZlMDQzZjNjZGE5ZGIzOGY0OnQ6VDpO>.
 We require you to either (1) present written permission from the copyright holder to publish these figures specifically under the CC BY 4.0 license, or (b) remove the figures from your submission:
 a . You may seek permission from the original copyright holder of Figure 7 to publish the content specifically under the CC BY 4.0 license.
 We recommend that you contact the original copyright holder with the Content Permission Form (<https://url.avanan.click/v2/___http://journals.plos.org/plosone/s/file?id=7c09/content-permission-form.pdf___.YXAzOmNhbGFjYWRlbXk6YTpnOjA1NmVlZTA1ZDJmMzJiMTZkOGU1NDQ1YWYzMTc0OTZiOjY6NmQ0MDo4NzJiMmZlMDQ5OGVlM2E1MTViOTViZTFjYTk1OTRiMTM3MTgzNzNlMGE3NWYxNGVhNzk0YTdmYzk0ZDU5MDUxOnQ6VDpO>) and the following text:
“I request permission for the open-access journal PLOS ONE to publish XXX under the Creative Commons Attribution License (CCAL) CC BY 4.0 (<https://url.avanan.click/v2/___http://creativecommons.org/licenses/by/4.0/___.YXAzOmNhbGFjYWRlbXk6YTpnOjA1NmVlZTA1ZDJmMzJiMTZkOGU1NDQ1YWYzMTc0OTZiOjY6MGRlODpkNjBjNDI0YTlkYTg0MDI0MTIwODdiN2JiYzE3MzNiMWVhM2QxMjEyN2IxNDM3OWFiZjNlZjA0MjA4OGQyZjAxOnQ6VDpO>). Please be aware that this license allows unrestricted use and distribution, even commercially, by third parties. Please reply and provide explicit written permission to publish XXX under a CC BY license and complete the att ached form.”
 Please upload the completed Content Permission Form or other proof of granted permissions as an "Other" file with your submission.
 In the figure caption of the copyrighted figure, please include the following text: “Reprinted from [ref] under a CC BY license, with permission from [name of publisher], original copyright [original copyright year].”
 b. If you are unable to obtain permission from the original copyright holder to publish these figures under the CC BY 4.0 license or if the copyright holder’s requirements are incompatible with the CC BY 4.0 license, please either i) remove the figure or ii) supply a replacement figure that complies with the CC BY 4.0 license. Please check copyright information on all replacement figures and update the figure caption with source information. If applicable, please specify in the figure caption text when a figure is similar but not identical to the original image and is therefore for illustrative purposes only.
The following resources for replacing copyrighted map figures may be helpful:
 USGS National Map Viewer (public domain): <https://url.avanan.click/v2/___http://viewer.nationalmap.gov/viewer/___.YXAzOmNhbGFjYWRlbXk6YTpnOjA1NmVlZTA1ZDJmMzJiMTZkOGU1NDQ1YWYzMTc0OTZiOjY6OTk3NzplZmMwZDcxMTJhYWY0ZGNlMjM3YjBmMzQ3MzZjNzE3> NGRkMGJiM2ZkYmNiZTEzMWMzNTMxMDA0OWZlYmU4YmY5OnQ6VDpO
The Gateway to Astronaut Photography of Earth (public domain): <https://url.avanan.click/v2/___http://eol.jsc.nasa.gov/sseop/clickmap/___.YXAzOmNhbGFjYWRlbXk6YTpnOjA1NmVlZTA1ZDJmMzJiMTZkOGU1NDQ1YWYzMTc0OTZiOjY6ZjQ3NDoyYjc2OGE3NzBhNjEzY2QzMDEwNzUxMjEyMWYxYzFlZGE2YTdkYjI2MTYwYjYwM2ZiYmUwOGU2NzEzYWM5MDBlOnQ6VDpO>
Maps at the CIA (public domain): <https://url.avanan.click/v2/___https://www.cia.gov/library/publications/the-world-factbook/index.html___.YXAzOmNhbGFjYWRlbXk6YTpnOjA1NmVlZTA1ZDJmMzJiMTZkOGU1NDQ1YWYzMTc0OTZiOjY6YjM3ZToyZjI0OTc0OTJkY2JjYzgzZTNjY2QyZGYxMTRjM2E5YTE0NTVjMzYyYzlhOWQzMmY5M2FjMGJkZDQzYmM3M2FhOnQ6VDpO> and <https://url.avanan.click/v2/___https://www.cia.gov/library/publications/cia-maps-publications/index.html___.YXAzOmNhbGFjYWRlbXk6YTpnOjA1NmVlZTA1ZDJmMzJiMTZkOGU1NDQ1YWYzMTc0OTZiOjY6YmQ2NDoyOGE3NzQwNzkzMzdjMGFiNjliNTc1N2YxNTg3ZGI2NWFjYTQyMDlhZTM3ZmRlY2E1NzE3YmIxNmY0MmYyYmRmOnQ6VDpO>
NASA Earth Observatory (public domain): <https://url.avanan.click/v2/___http://earthobservatory.nasa.gov/___.YXAzOmNhbGFjYWRlbXk6YTpnOjA1NmVlZTA1ZDJmMzJiMTZkOGU1NDQ1YWYzMTc0OTZiOjY6NjlmZjphZmQ4ODU2MGE4MDU5M2U3NDNjMjZiOTI1OTBjMjFiYzdkYzczNTRmNjcyMWEyYzcyMjE2MjllNWIxZDVkZjM2OnQ6VDpO>
Landsat: <https://url.avanan.click/v2/___http://landsat.visibleearth.nasa.gov/___.YXAzOmNhbGFjYWRlbXk6YTpnOjA1NmVlZTA1ZDJmMzJiMTZkOGU1NDQ1YWYzMTc> 0OTZiOjY6MjFiMzo3OGRmNWUzZjUxODlhMmIwMmM5ZjFlZjE3YWI3MzIxMTY1ZDg0MWY4YjliNDMyYTZlOGEwZTI1MmE3NjgxYWNkOnQ6VDpO
USGS EROS (Earth Resources Observatory and Science (EROS) Center) (public domain): <https://url.avanan.click/v2/___http://eros.usgs.gov/%23___.YXAzOmNhbGFjYWRlbXk6YTpnOjA1NmVlZTA1ZDJmMzJiMTZkOGU1NDQ1YWYzMTc0OTZiOjY6NzU4NzpkNDllZTYwOTQyZTgxZjA3M2U5M2Q1NGFjMzg5MTYzNzllYmNhMjdmMjdlZTUwMjM5YjJkNTNhMTlkZTIzNmZjOnQ6VDpO>
Natural Earth (public domain): <https://url.avanan.click/v2/___http://www.naturalearthdata.com/___.YXAzOmNhbGFjYWRlbXk6YTpnOjA1NmVlZTA1ZDJmMzJiMTZkOGU1NDQ1YWYzMTc0OTZiOjY6YjM0MjpjZjlmNDc2ZTU3M2E1NjM2ODRmYjEyZDVjODU0MDNhMzM5YTE1NGJkMTUxOGQwNzI3ZDM4MTUxMzdhMDdlZDY2OnQ6VDpO>

We added the following line to the caption for Figure 6: “Background map is included in the R package ggplot2 v.3.4.0” (line 282). It is most likely that this map is rendered from those available through [Natural Earth](https://www.naturalearthdata.com/about/terms-of-use/), which is an open-source mapping platform. We did not upload any other maps.

7. Please remove your figures from within your manuscript file, leaving only the individual TIFF/EPS image files, uploaded separately. These will be automatically included in the reviewers’ PDF.

We have removed the images from the manuscript file.

8. Please include captions for your Supporting Information files at the end of your manuscript, and update any in-text citations to match accordingly. Please see our Supporting Information guidelines for more information: <https://url.avanan.click/v2/___http://journals.plos.org/plosone/s/supporting-information.___.YXAzOmNhbGFjYWRlbXk6YTpnOjA1NmVlZTA1ZDJmMzJiMTZkOGU1NDQ1YWYzMTc0OTZiOjY6ZGFjMDpkY2E3N2U2NjU4MDJiZjU2NjZjOGZmOTY0MGU5ZGM2OGFkMDZhYjljNWQ3NTk5YTUxMDM5OTI3ZjI2NzcxMTk1OnQ6VDpO>

We have moved supplementary figure and table captions to the end of the manuscript and formatted them as directed.

9. Please review your reference list to ensure that it is complete and correct. If you have cited papers that have been retracted, please include the rationale for doing so in the manuscript text, or remove these references and replace them with relevant current references. Any changes to the reference list should be mentioned in the rebuttal letter that accompanies your revised manuscript. If you need to cite a retracted article, indicate the article’s retracted status in the References list and also include a citation and full reference for the retraction notice.

We have added some suggested references regarding range shifts, in addition to a few others throughout. We have also removed any references that we no longer cite in the manuscript. We updated the works cited accordingly.

Reviewers' comments:

Reviewer's Responses to Questions

**Comments to the Author**

1. Is the manuscript technically sound, and do the data support the conclusions?

The manuscript must describe a technically sound piece of scientific research with data that supports the conclusions. Experiments must have been conducted rigorously, with appropriate controls, replication, and sample sizes. The conclusions must be drawn appropriately based on the data presented.

Reviewer #1: Partly

Reviewer #2: Yes

Reviewer #3: Yes

2. Has the statistical analysis been performed appropriately and rigorously?

Reviewer #1: N/A

Reviewer #2: Yes

Reviewer #3: N/A

3. Have the authors made all data underlying the findings in their manuscript fully available?

The [PLOS Data policy](https://url.avanan.click/v2/___http://www.plosone.org/static/policies.action%23sharing___.YXAzOmNhbGFjYWRlbXk6YTpnOjA1NmVlZTA1ZDJmMzJiMTZkOGU1NDQ1YWYzMTc0OTZiOjY6OTliMjozZWJmM2UxMGJlNzFmYjZhNzRjNTEyZDU2MTNkMTQ3Zjg1MTc3OWFhMjg5NzRiZmYyZmFkZDZmMmYwZTE1NWUzOmg6VDpO) requires authors to make all data underlying the findings described i n their manuscript fully available without restriction, with rare exception (please refer to the Data Availability Statement in the manuscript PDF file). The data should be provided as part of the manuscript or its supporting information, or deposited to a public repository. For example, in addition to summary statistics, the data points behind means, medians and variance measures should be available. If there are restrictions on publicly sharing data—e.g. participant privacy or use of data from a third party—those must be specified.

Reviewer #1: Yes

Reviewer #2: Yes

Reviewer #3: Yes

4. Is the manuscript presented in an intelligible fashion and written in standard English?

PLOS ONE does not copyedit accepted manuscripts, so the language in submitted articles must be clear, correct, and unambiguous. Any typographical or grammatical errors should be corrected at revision, so please note any specific errors here.

Reviewer #1: Yes

Reviewer #2: Yes

Reviewer #3: Yes

5. Review Comments to the Author

Please use the space provided to explain your answers to the questions above. You may also include additional comments for the author, including concerns about dual publication, research ethics, or publication ethics. (Please upload your review as an attachment if it exceeds 20,000 characters)

Reviewer #1: The study reports the species identification for a series of Hermissenda specimens found on debris that crossed the Pacific following the 2011 tsunami in Japan. The complexities regarding the three pseudocryptic species of Hermissenda and the fact that all adults of species identified on tsunami debris to date have originated in the eastern Pacific add a layer of interest to confirming the species IDs. The findings are clear cut: the specimens seem to be eastern Pacific species. Unfortunately, the manuscript provides a rather convoluted and disjointed presentation of this finding. Moreover, related investigations with regard to phylogenetic analyses of the three species and range limits based on iNaturalist could have some improvements and be better incorporated into single narrative for the whole manuscript.
Major Comments

1. Overall, I suggest that the manuscript could be considerably shortened and the flow of ideas made considerably simpler. Currently, the manuscript repeatedly raises the possibility that the Hermissenda could have rafted from Japan, when no evidence supports this, there are several unnecessary tangential paragraphs or sections, and there could be better separation of material between introduction, methods, results and discussion. I suggest the following outline for the manuscript, which I think we would be clearer: Hermissenda was found on debris from Japan. Because of the timing of when they were identified and origin of the debris, they could be any one of the 3 pseudocryptic species. This raises three questions. First, what species were they? Second, since only sequence data are available for IDing the specimens, does the addition of these sequences to molecular phylogenies causes any issues with the clear molecular separation of the 3 pseudocryptic Hermissenda species? Lastly, since it turns out both opalescens and crassicornis are present on the debris, what is the latest evidence on the ranges of those species? Since there is plenty of overlap in range, and these nudibranchs are fast growing generalist feeders, it seems quite feasible that planktonic larvae of both species would have been able to settle and grow to adulthood in the biofouling community on the debris while it was in eastern Pacific waters. All of the various bits for this outline are currently in the manuscript, but they are not laid out in this order and are interrupted by intervening tangential ideas. I suggest revising the entire manuscript to be organized around this outline, starting with a revised title, which could be far more informative of what the major finding is. Suggestion: A trans-Pacific rafting community may have received Hermissenda immigrants from the destination habitat.

We have taken these ideas into account and have rearranged the content of our introduction section and revised the title. We have clarified the use of molecular phylogeny to address our question of identifying the nudibranchs on vessel JTMD-BF-356 (lines 126–138).

2. At the moment, there seems to be some circularities in the way the data is considered. The new specimens from the vessel debris are from an unknown population location. No morphological data on the cerata are presented. Thus, they can only be identified using COI sequence similarity with previously identified specimens from other studies, with no other corroborating evidence (see below re radula dentition). Given this logic flow, it is tautological to then consider the species delineations according to sequences. Proper evidence for that task requires morphological and sequence data for all individuals. I suggest interpretation of the phylogenetic trees and haplotype networks beyond IDing the specimens should be limited to assessing whether the new sequences cause any meaningful change to the clades that correspond to each of the species, as shown in Lindsay and Valdes. This approach may have been the intention from the outset, but that is not how the explanations come across in lines 282-290 or so. Instead the arguments appear to be taking the sequences as evidence supporting species delineation – which is a circular argument since the species IDs were determined using those sequences.

Our goal was to figure out where our specimens fell within the existing phylogeny of *Hermissenda*, rather than interrogate the *Hermissenda* species relationships. We have walked back some language throughout the paper regarding species delimitation and moved the related analyses to the supplementary section. We decided to keep these because we have added a significant number of *Hermissenda* sequences to the GenBank repository that are now all considered together in the phylogeny of *Hermissenda*; other researchers may see these findings as interesting, even if they are not tied to our central aims.

3. No external morphological data is presented in the results. Yet in the discussion, photographs consistent with crassicornis are mentioned. This is highly problematic, both because these were apparently not treated as useful results, but also because it contradicts the molecular identification of some specimens as opalescens. The one picture provided in the methods does not have adequate resolution to assess white tips or lines on the cerata. Surely this data should be thoroughly addressed in the results, and the discrepancy needs to be discussed? At least a couple of the specimens in the methods figure appear to have widely separated groups of cerata, possibly consistent with the morphology of emurai? Note that depending on how this aspect is dealt with it may well change how the molecular data needs to be interpreted. In particular, which sequences in the phylogenetic trees that are assigned to the opalescens clade have pictures showing white stripes? Do they cluster together? Does removing these ambiguous specimens change the node support values?

In the introduction, we added a statement making it clear that all the evidence we had to work with were the single photograph of the cluster of nudibranchs and the nudibranch specimens themselves, none of which is associated with a particular individual in the photograph (Fig 2). Thus, we could not include a meaningful discussion of external morphology. We have removed the sentence about the lines on the cerata in the photograph.

The two specimens that appear to have more widely spaced ceratal clusters are floating on surface tension and their posture changes as a result. However, their ceratal color is consistent with *H. opalescens* not *H. emurai*. None of our specimens cluster with *H. emurai* in the molecular analysis.

4. For the range limits, I found the exclusive use of iNaturalist data with no quality control to be troublesome. I am most familiar with the northward end of the range, and a quick check revealed that the 2 most northerly locations for opalescens (Alaska, Haida Gwaii) were a single specimen photographed 4 times that does seem to have white tips and a specimen that could easily be a Dendronotus not a Hermissenda. Tellingly, the Haida Gwaii specimen is not shown in Fig 7, and was changed to Dendronotus following my query about its veracity! Moving a northward range boundary from Vancouver Island (where there are many iNaturalist observations and the evidence from Merlo et al) to Alaska based on such scant and dubious evidence seems to far over reach usual standards. I suggest some thorough checking of the images from the extremes of the distributions, and some systematic sub sampling of all the iNaturalist images to have some idea of the quality of that data. A quick search revealed a range of studies that can give guidance on appropriate methods here. Regardless, I suggest much more cautious language should be used regarding the range limits of the species.

Our method of mapping *Hermissenda* occurrences involved taking GBIF observations, which include Research Grade iNaturalist observations, and plotting them on a map of the eastern Pacific. We have added a statement in the discussion regarding uncertainties in iNaturalist identifications and removed some data accordingly (lines 328–333) and tempered our language regarding range throughout the manuscript. The northernmost *H. opalescens* observations from Sitka, AK were doubtful, so removing those changed the northernmost occurrence of *H. opalescens* and the mean latitude. We updated Figure 6 accordingly.

5. It is surprising that the manuscript does not engage with the studies of range shifts among nudibranchs and other taxa along the Pacific coast of North America. In particular, some discussion of whether shifts thought to be associated with marine heat wave ten years ago may be persisting. Some relevant literature (and there may be others – I suggest cross referencing from these studies):
Goddard, J., Treneman, N., Pence, W., Mason, D., Dobry, P., Green, B., and Hoover, C. 2016. Nudibranch range shifts associated with the 2014 warm anomaly in the Northeast Pacific. Bulletin of the Southern California Academy of Sciences 115(1): 15–40.
Goddard, J.H.R., Treneman, N., Prestholdt, T., Hoover, C., Green, B., Pence, W.E., Mason, D.E., Dobry, P., Sones, J.L., Sanford, E., Agarwal, R., McDonald, G.R., Johnson, R.F., and Gosliner, T.M. 2018. Heterobranch Sea Slug Range Shifts in the Northeast Pacific Ocean associated with the 2015-16 El Niño. PROCEEDINGS OF THE CALIFORNIA ACADEMY OF SCIENCES 65(3): 25.
Sanford, E., Sones, J.L., García-Reyes, M., Goddard, J.H., and Largier, J.L. 2019. Widespread shifts in the coastal biota of northern California during the 2014–2016 marine heatwaves. Scientific reports 9(1): 4216.

We have added the suggested references to our discussion of overlapping ranges of *H. crassicornis* and *H. opalescens* (line 336).

6. The dentition data has far too limited a sample size. Given the uncertainties, as currently acknowledged in the discussion, I suggest removing all reference to this aspect of the study in the methods and results. A single line in the discussion would suffice, referencing unpublished preliminary results, suggesting that the dentition information in Lindsay and Valdes may need to be re-examined with a comprehensive survey.

We have minimized our discussion of these results (lines 247–252) and their minute implications in the discussion (lines 319–324).

Minor comments
7. The first half of the first introduction paragraph (lines 51 – 59) seems irrelevant for this study.

We have reduced the discussion of nudibranch diet in this introduction section.

8. The explanation regarding plankton life cycles and rafting is confusing (lines 61 – 62). Isn’t the point here that there are two possible mechanisms of dispersal for Hermissenda since it has planktonic larvae and can live in biofouling communities?

We have edited this section and broadened the discussion of nudibranch natural history (lines 50–56).

9. The paragraph on invasion and dispersal (line 86+) seems far too broad and convoluted. The key point for this study is simple: nudibranch dispersal can be via planktonic stages or via rafting.

We have removed much of this paragraph and moved up some of the lines to where they are more relevant (lines 75–80).

10. Line 153 – “species” should probably be “specimens”?

Corrected.

11. Line 187+ – the iNaturalist methods should be in a separate paragraph

We have made the GBIF mapping methods into a separate paragraph, now lines 202–204.

12. The first two paragraphs in the results (lines 195 – 207) do not seem to be results from this study, or at least that is what seems to be the suggestion from a related paragraph in the introduction (lines 107 -115). The information in the results seems to be extra detail from Carlton et al. publications? If they are new results, the methods by which they were achieved need to be included in the methods section. All of this information needs to more clearly explained, and only in the appropriate section (intro, methods, results, or discussion).

We have moved these lines to the introduction, now lines 67–75. These lines were included to provide further context to the contents of vessel JTMD-BF-356.

13. The phylogeny figures are illegible because font sizes are far too small. These raw images should be included only as supplementary figures. Alternatively, they need to be presented with changed fonts sizes, line sizes/colours and/or additional annotations to make the key points readable – that all sequences sort into two of the three clades, and they do not cause any substantive changes to the clade relationships shown by Lindsay and Valdes.

We have decided to keep the size of the phylogeny but have added further annotations as suggested and confirmed in the discussion that there are no substantive changes to the Lindsay and Valdés clade relationships (lines 307–308).

14. The bolded conclusion on line 281 seems backward. The two species are not in Japan so why would they raft? Instead, the identification of the specimens as opalescens and crassicornis means that emurai did not raft. See comment 1 above.

We have removed this sentence.

15. Line 319 – 320. Another case of rafting be presented as a possibility when it’s already been concluded that they did not raft. See comment 1 above.

We have removed this sentence.

16. Line 323+ This paragraph on diet seems mostly irrelevant. Hermissenda is a generalist that feeds in biofouling communities in its native range. Given many similar species are present in northewestern Pacific biofouling communities, it is certainly plausible that they found adequate prey supply on the debris.

We have kept this paragraph because it provides further context for the process by which the nudibranchs likely occupied the raft.

17. Line 350 food preferences have already been studied:
Hoover, R.A., Armour, R., Dow, I., and Purcell, J.E. 2012. Nudibranch predation and dietary preference for the polyps of Aurelia labiata (Cnidaria: Scyphozoa). Hydrobiologia 690(1): 199–213. doi:10.1007/s10750-012-1044-x.

We ended up removing this line and references to past or future study of food preferences.

18. Line 355+. The information that no adult eastern Pacific species have been found on any other pieces of debris to date is compelling. This should probably be a key point in the introduction. Saving that to the very last paragraph of the discussion is counter productive.

We have decided to keep this information’s position to round out the end of the paper by bringing back to the larger discussion of the marine debris and the nudibranchs’ divorce from the story of post-tsunami dispersal.

19. The final paragraph makes an interesting point – that the alternative possibility of opalescens and crassicornis both being present in Japan is not inconsistent with the data. I suggest avoiding the tangential points regarding multiple populations and adults being washed away. Isn’t the point that if this possibility did happen, it would require one (or more) of the following: unusually short transit time from West to East, adult survival of 1+ years (despite evidence of shorter lifespans), or one or more generations, made possible by the Hermissenda living inside a tank which could have reduced dispersal by the planktonic larvae?

We have revised this paragraph and removed the suggestion that *H. opalescens* and *H. crassicornis* are in Japan, due to its highly unlikely nature.

Reviewer #2: Montana et al. Hermissenda JTMD


Montana et al. address the origins of two Hermissenda species recovered from an approximately 8 m length of the bow of a Japanese aquaculture fish tender that arrived on the Oregon coast in 2015, four years after the 11 March 2011 Japanese earthquake and tsunami. Montana et al. use genetics and morphology to test whether these Japanese Marine Tsunami Debris (JTMD) specimens were the only known Japanese Hermissenda (H. emurai) or included other, eastern Pacific species. Their question is particularly interesting because no other mature nearshore/intertidal benthic invertebrates of the eastern Pacific were discovered on any of the other 633 JTMD items examined. Their detailed analyses, figures and tables of external morphology, life history development, SEM, biogeography and DNA sequencing are convincing. Their exposition overall is excellent and their results are original. This MS would be a worthy contribution to PLOS ONE.

Minor comments:

Montana et al. ask in their title: “Why didn’t the nudibranch cross the ocean?” but their question is “whether” the nudibranchs crossed the ocean. Their analyses reveal that only the eastern Pacific H. crassicornis and H. opalescens were present. Those Hermissenda, unknown in Japan, appear unlikely to have crossed the ocean. Their conclusion that these nudibranchs were far more likely to have settled and grown to maturity on the wreck after it reached the eastern Pacific, after it crossed the ocean, is convincing. Two other apparently eastern Pacific nudibranchs were recovered from the wreck: Dendronotus venustus and Eubranchus rustyus (visually identified) and E. rusticus confirmed also by molecular data.

We have changed the title to be more inclusive of the non-*Hermissenda* eastern Pacific nudibranchs and make the aim of our study more clear.

Montana et al. conclude that these nudibranch pose a “striking conundrum” when considering no other of the 633 JTMD objects examined between 2012 and 2017 supported reproductively mature eastern Pacific invertebrates. More like a “challenge”? This wreck (JTMD item BF-356) was different from all other JTMD, that was launched on the afternoon of 11 March 2011. BF-356 was highly likely to have broken away from its aft section long (years) after 2011. An emphasis that the initial timing and the ocean crossing history and eastern Pacific history of BF-356 was different from all other JTMD objects would be nice to include in the analyses. Is it possible, for example, that where something drifts and lands continues to be influenced by when it was released, even years after it is released? Moreover, Montana et al. do not seem impressed that all four of the recovered nudibranchs were reproductively mature and very likely from the eastern Pacific. The exceptions were not confined to two species. Lighting struck four times, not twice. Why only nudibranchs?

Please see responses to comments 18 and 19 above. We have emphasized this unique situation in the final paragraph.

General comments:
Typo at end of line 6

We were not sure what this comment was referring to because the original line 6 was authors’ names.

Typo line 364

We have removed this line.

Reviewer #3: Please, read the attached review carefully. Improvements and corrections are easy to do and they shouldn't take much time (if sequences of 18S, 16S and H3 markers are available). Some paragraph of the abstract was complicate to understand and nees to be re-written.

**GENERAL REMARKS**:

This manuscript is addressed contribute for a better understanding of the effects of the tsunami happened in 2011 in Japan concerning the transportation/invasion of organisms from western to eastern Pacific coasts. Thus, the study is focussed on the species of the aeolid nudibranch genus *Hermissenda* (*H. crassicornis* and *H. opalescenes*) found on a vessel from Iwate Prefecture (later designated as JTMD-BF-356) discovered at the coast of Oregon in april 2015. A good number of non native invertebrate species were found and reported previously on the surface of this vessel. Thus, among the nudibranch individuals found, stand out two species: *Dendronotus venustus* and *Eubranchus rustyus*, both only known from the western Pacific. However, the individuals of the genus Hermissenda belongs to H. crassicornis and H. opalescens, both known to date to eastern Pacific only. No individuals of *H. emurai* (from western Pacific) were found among the vessel *Hermissenda* individuals. Then, two hypothesis arise to try to explain the presence of the above species: or they were settled after the arrival of the vessel or both species are present in Japanese coasts, but this presence were unnoticed to date.

Taxonomical idendification of *Hermissenda* species is confirmed by molecular phylogenenetic and haplotype studies, complemented by a delimitation species análisis.

In general terms, I agree with the conclusions of the manuscript. Nevertheless, I have several concerns that should be corrected/clarified/discussed before the manuscript could be published (see below).

**PARTICULAR REMARKS:
 Title:** place “Bergh, 1879” after *Hermissenda*. Moreover, the date of the genus is 1879, instead

1878 (see https://www.marinespecies.org/aphia.php?p=tax details&id=367881)

We have changed the title but changes the reference to Bergh, 1879 when referring to *Hermissenda* the first time.

**Abstract:**

Lines 42-44: “Our phylogenetic tree...*H. opalescens* and *H. emurai*”. I don’t understand the meaning of this paragraph. Please, check and re-write it.

As assessing species relationships of *Hermissenda* was not the main aim of this study, we have removed this line.

Lines 41 and 44-46: I think that the reference to iNaturalist should be linked with the final paragraph (44-46). Please, re-write again.

We have removed this final sentence of the abstract.

**Introduction:**

Line 57: modify as “...Megina & Cervera, 2003; Nakano & Hirose, 2011) or even eggs of other nudibranchs or fishes (Todd, 1981; Calado & Urgorri, 2002).”

We have added these references as suggested.

Line 61: modify as “(Hadfield, 1963; Todd, 1981)”.

We have added the Todd, 1981 reference as suggested.

Line 82: change “family *Hermissenda*” by “genus *Hermissenda*”.

We have edited this error, now lines 101–102.

**Methods:**

a) Vessel JTMD-BF-356

Line 136: change “(Carlton et al. 2017; Carlton et al. 2018...” by “Carlton et al., 2017, 2018,...”

We have reformatted the in-text citations according to journal specifications.

b) Molecular analyses of nudibranchs

Why the authors have used the COI sequences in their analyses only? Lindsay and Valdés (2016) used two mitochondrial (COI, 16S) and two nuclear markers (18S, H3) and their resulting phylogeny has its nodes well supported. Their phylogenetic hypothesis is the same of that presented by Montana et al (this manuscript), but in this last case only the monophyly of the genus Hermissenda is well supported, although the suggested/pointed rela�onships amomg species are similar to that of Lindsay and Valdés (2016). I suggest to inclued more molecular markers to get well supported nodes in the phylogene�c tree.

Since the scope of this study was not to relimit the species but to identify the nudibranchs found on vessel JTMD-BF-356 and set them in the greater phylogeny of *Hermissenda*, we only sequenced COI. We no longer have the capacity to include other molecular markers.

On the other hand, values to consider nodes well supported for each analysis are not indicated. Nor in the text nor the figure captions of figs 3 and 4. Please, do it.

We have added that by low support we mean node values <95 or 0.95, depending on the inference method (line 212).

**Results:**

a) JTMD-BF-356 biota
 Line 196: change “fish” by “fishes”.

We have corrected this.

Line 196: change “Carlton et al. 2017; Carlton et al. 2018” by “Carlton et al., 2017, 2018”.

We have reformatted the in-text citations according to journal specifications.

b) Molecular analyses and phylogenetic relationships of *Hermissenda* Please, see what I have remarked in the Methods section concerning to the molecular
 analyses of nudibranch that refers to the support of the clades.

We include the more support values in the report of the results of our phylogeny (lines 214–216).

Lines 235-240: the authors refers to the number of denticles of the radular central cusp of the different species of *Hermissenda*, and also refers to the infomation supplied by Lindsay and Valdés (2016). However, I think the authors of the manuscript have been confused. Lindsay and Valdés (2016) didn’t find significant radular differences among the rádula of the three species of *Hermissenda*. However, they found slightly morphological differences among the denticulaon of the mas�catory border of the jaws. Then, part of this paragraph needs to be rethought and rewritten again.The authors supply radular information, but not that concerning to jaws that could be relevant (to differentiate species in this genus) according to Lindsay and Valdés (2016) stated.Nevertheless, in my opinion, the number of examined specimens both by Linsay and Valdés (2016) and in the present manuscript are are not enough to have a clear idea about the relevance of the differences of the radulae and jaws among *H. crassicornis*, *H. opalecens* and *H. emurai*. I would check this much beter...and the authors have enough material to carry on this.

We have revised this section to minimize discussion of morphology. Please see lines 247–252.

There are no differences among the reproductive systems of the three species? Lindsay and Valdés (2016) didn’t refer to this.

We did not collect data on the reproductive systems of the species.

Line 240: “Lindsay and Valdés, 2016”. Here the authors use “and” instead “&”. Please, check all the text to homogenize this in all references

We have corrected this.

**Discussion:**

Lines 296-306: please, see what I have remarked in the above section (Results) concerning the study of radula and jaws.

We have revised our discussion of morphology. Please see lines 247–252, as above.

Lines 323-339: this paragraph is very interesting because concerns the feeding and larval biology. Thus, the authors refers to Kincaid and de Rivera (2021) report that *H. crassicornis* feeds preferentially on the indigenous ascidian *Distapla occidentalis* than to the non-indigenous *Botrylloides violaceus* or *B. schlosseri*. Surprisingly, Kincais and de Rivera overlooked the previous paper of Megina et al (2007) describing the trophic strategies of *Hermissenda crassicornis*. The individuals attributed to *H. crassicornis* were collected south to San Francisco Bay and Monterey. So, probably were specimens of *H. opalescens*, according to the range proposed in Lindsay and Valdés (2016). Megina et al (2007) observed that the main prey of *H. “crassicornis”* was *Aplidium “solidum”*. However, the nomenclature of this species was revised Brune� (2007) and the Californian called “*solidum*” is *A. kottae* Riter and Forsyth, 1917. It would be very interesting/useful to know if *Aplidium kottae* was setled on the JTMD- BF-356 vessel. However, no detailed information on Tunicates is included in Carlton et al 2017 and 2018. Maybe Prof. James T. Carlton could have this information.

*Aplidium kottae* was not on the species list in the supplementary section of Carlton et al., 2017 or 2018. JTC has confirmed that there was not any *Aplidium* on the vessel, and essentially no ascidians.

In any case, both *Hermissenda crassicornis* and *H. opalescens* seem to prey on indigenous tunicates preferently (*Distapla occidentalis* and *Aplidium kottae*, respectively), but not exclusively. Moreover, at least *H. opalescens* can feed on hydrozoans or antozoans probably as a source of nematocyst (Megina et al, 2007). So, I think that it is much probable that *Hermissenda* populations of the JTMD-BF-356 vessel settled after its arrival to the Oregon coast. But, I agree that further investigations have to be carried out to exclude the alternative hypothesis (presence of *H. crassicornis* and *H. opalescens* in Japanese coasts).

Thank you for providing this context to *Hermissenda* diet.

**Acknowledgements:**

Line 386: change “Hermissenda” by “*Hermissenda*” (cursive)

Fixed.

*References cited in this review and not included in the Bibliography*:

- Brune, R. 2007. Nomenclatural Acts: homonymy in the Ascidiacea (Tunicata) and proposed nomina nova. *Zootaxa*, 1613: 67-68.

- Calado, G. and Urgorri, V. 2002. A new species of *Calma* Alder & Hancock, 1855 (Gastropoda: Nudibranchia) with a review of the genus. *Journal of Molluscan Studies*,68: 311-317.

- Megina, C., Gosliner, T.M. and Cervera, J.L. 2007. The use of trophic resources by a generalist eolid nudibranch: *Hermissenda crassicornis* (Mollusca: Gastropoda). *Cahiers de Biologie Marine*, 48: 1-7

- Todd, C.D. 1981. The ecology of nudibranchs molluscs. *Oceanography and Marine Biology: An Annual Review*, 19: 131-234.
